# Association between non-adherence to fish oil or placebo as a risk factor of transition to psychosis in ultra-high-risk individuals in the NEURAPRO study

**DOI:** 10.1177/00048674251361758

**Published:** 2025-08-25

**Authors:** Monika Schlögelhofer, Ashleigh Lin, Connie Markulev, Miriam R Schäfer, Patrick D McGorry, Barnaby Nelson, Rebekah Street, Nilufar Mossaheb, Stefan Smesny, Ian B Hickie, Gregor Berger, Eric YH Chen, Lieuwe de Haan, Dorien H Nieman, Merete Nordentoft, Anita Riecher-Rössler, Swapna Verma, Andrew Thompson, Alison R Yung, G Paul Amminger

**Affiliations:** 1 Department of Child and Adolescent Psychiatry, Medical University of Vienna, Vienna, Austria; 2 BioPsyC—Biopsychosocial Corporation, Non-Profit Association for Research Funding Ltd., Vienna, Austria; 3 School of Population and Global Health, The University of Western Australia, Perth, WA, Australia; 4 Orygen, National Centre of Excellence in Youth Mental Health, Melbourne, VIC, Australia; 5 Centre for Youth Mental Health, The University of Melbourne, Melbourne, VIC, Australia; 6 Clinical Division of Social Psychiatry, Department of Psychiatry and Psychotherapy, Medical University of Vienna, Vienna Austria; 7 University Hospital Jena, Jena, Germany; 8 Brain and Mind Centre, The University of Sydney, Camperdown, NSW, Australia; 9 Department of Child and Adolescent Psychiatry and Psychotherapy, Psychiatric Hospital, University of Zürich, Zürich, Switzerland; 10 Department of Psychiatry, School of Clinical Medicine, LKS Faculty of Medicine, The University of Hong Kong, Hong Kong; 11 Department of Psychiatry, Amsterdam UMC, The Netherlands; 12 Psychiatric Centre Bispebjerg, Copenhagen, Denmark; 13 Psychiatric University Clinics Basel, Basel, Switzerland; 14 Institute of Mental Health, Singapore, Singapore; 15 Division of Mental Health and Wellbeing, Warwick Medical School, University of Warwick, Coventry, UK; 16 Deakin University, Melbourne, Australia

**Keywords:** Non-adherence, study medication, omega-3 polyunsaturated fatty acids (omega-3 PUFAs), ultra-high risk, psychosis

## Abstract

**Objective:**

Non-adherence is an important factor in clinical trials, which has not been investigated in people at ultra-high risk (UHR) of developing a first episode of psychosis.

**Methods:**

Exploratory analysis of data from NEURAPRO, a multicenter, placebo-controlled trial of long-chain omega-3 polyunsaturated fatty acids (omega-3 PUFAs) in 304 individuals at UHR. We examined correlates of non-adherence with study medication (omega-3 PUFAs or placebo), including patient, illness and treatment factors, plus transition to psychosis. Non-adherence was defined as <75% study medication intake over 6 months and, post hoc, by the number of returned pills.

**Results:**

Of 285 randomized participants with baseline fatty acid data, 163 (57.2%) were non-adherent. In univariate analyses, non-adherence was associated with baseline omega-3 index, pre-baseline duration of untreated symptoms, smoking, cannabis use, lower baseline Social and Occupational Functioning Assessment Scale, Global Functioning: Social and Role Scale scores and transition to psychosis. Transition to psychosis risk was significantly lower in the adherent than non-adherent group (4.2%, 95% CI = 0.7–7.7% vs 17.3%, 95% CI = 10.4–24.2%), Kaplan–Meier Log-rank test, chi-square = 10.675, *p* = 0.001), independent of omega-3 PUFA treatment status. Similarly, Cox regression analysis, covarying for the aforementioned factors significantly associated with non-adherence, also revealed non-adherence as an independent predictor of transition to psychosis (*B* = 1.452, *p* = 0.005). Finally, non-adherence was also significantly associated with transition to psychosis, even when defining non-adherence by number of returned pills.

**Conclusion:**

Non-adherence predicted a higher risk of progressing to psychosis in UHR individuals. Further studies are needed to better understand factors contributing to non-adherence and how non-adherence is related to transition to psychosis.

## Introduction

Non-adherence to medication is generally defined as the extent to which patients do not take medications as prescribed (Hickling et al., 2018; Osterberg and Blaschke, 2005). Since medication intake is essential for its effectiveness, non-adherence is a major problem in medicine and clinical trials. Non-adherence is especially problematic for people with psychosis since it not only prevents treatment from achieving its intended effect, but suboptimal adherence is also related to higher rates of relapses, reduced quality of life, poorer symptomatic outcome and longer duration of hospitalization (Kane et al., 2013). Furthermore, non-adherence can confound the interpretation of clinical trial results, leading to an underestimation of the efficacy of tested interventions.

The measurement of non-adherence is complicated by a lack of a universally agreed cut-off or standard for what constitutes adequate adherence. Medication intake as prescribed for 75–80% of time has generally been considered an acceptable level of adherence (Julius et al., 2009; Velligan et al., 2007, 2009). To achieve the same statistical power at a lower adherence rate, more participants are needed (e.g. a 50% greater sample, given a 20–30% study medication non-adherence) (Smith, 2012). An additional methodological difficulty is the reliable measurement of adherence. Adherence can be measured either directly or indirectly (Kane et al., 2013). Direct measures include concentration of a drug or its metabolite in blood or urine. Indirect methods include assessing clinical response, performing pill counts, ascertaining rate of refilling prescriptions, collecting patient questionnaires, using electronic medication monitors, smartphone medication adherence apps (e.g. smartphone apps to record videos of medication intake; (Dayer et al., 2013; Perez-Jover et al., 2019), measuring biomarkers, or asking the patient to keep a medication diary. Each measure has its advantages and disadvantages, but no method is without limitations or considered ideal (Osterberg and Blaschke, 2005).

Regardless of the method used, non-adherence seems to differ between people with psychiatric disorders and physical conditions (Cramer et al., 2003). People with psychiatric disorders have lower rates of adherence compared to people with physical disorders (Haddad et al., 2014; Perkins, 2002). For example, a systematic review of randomized and non-randomized studies by Cramer and Rosenheck (1998) spanning papers published between 1975 and 1996 showed that the adherence rate among people with physical conditions was the highest with 76% (range 40–90%), while people with psychiatric disorders had considerably lower adherence, which differed considerably across disorders. People with psychotic disorders had the lowest adherence rate at 58% (range = 24–90%), which was significantly lower than in other psychiatric disorders, for example, depression (65%; range = 58–90%). The latest systematic review (Sendt et al., 2015), which included 13 observational studies (*N* = 6235), reported adherence rates ranging from 47–95% in schizophrenia-spectrum disorders. A recent study (Hickling et al., 2018) concluded that 30–40% of first episode psychosis (FEP) patients became non-adherent within 6 months, increasing up to 50% at 1 year (Hickling et al., 2018; Klingberg et al., 2008).

Data about medication non-adherence and risk of transition to psychosis in individuals at clinical high-risk or ultra-high risk (UHR) for psychosis are scarce. A naturalistic study (Cornblatt et al., 2007b) of participants with attenuated psychotic symptoms treated with either antidepressants or second-generation antipsychotics (SGAs) reported a 44% transition rate to psychosis in the clinician-assigned SGA group compared to 0% in the antidepressant group. Further analyses revealed that the transitions to psychosis occurred only in adolescents who had become non-adherent to SGAs at least 6 months prior. However, because of the naturalistic study design and likely group difference at baseline, it is difficult to draw firm conclusions from this study.

The NEURAPRO randomized controlled trial (RCT) tested omega-3 polyunsaturated fatty acids (omega-3 PUFAs) versus placebo to prevent a first episode of psychotic disorder in UHR patients who received cognitive behavioral case management (CBCM) as background therapy (McGorry et al., 2017). Over 50% of participants in NEURAPRO where non-adherent based on returning >25% study medication capsules. Aims of this exploratory analysis were (1) to determine factors associated with non-adherence to study medication in NEURAPRO and (2) to test whether non-adherence predicted transition to psychosis. Since there was no significant difference in transition rates between the two treatment conditions (omega-3 PUFAS vs placebo), analyses were conducted in the entire sample. Based on the existing literature, we hypothesized that poor premorbid functioning and comorbid substance use would be associated with non-adherence. Moreover, we hypothesized that non-adherence would be associated with transition to psychosis.

## Methods

We conducted post hoc analyses in NEURAPRO, a multi-center, double-blind, placebo-controlled RCT to prevent/delay the onset of psychosis in participants at UHR, testing 6 months treatment with omega-3 PUFAs versus placebo, alongside CBCM (clinical trials.gov identifier: NCT00396643). Details of the method of this trial were previously reported by McGorry and colleagues (McGorry et al., 2017).

Measures included in this analysis were age, gender, body mass index (BMI), tobacco use and cannabis use. Baseline symptoms were measured with the Brief Psychiatric Rating Scale (BPRS) (Overall and Gorham, 1962), Scale for the Assessment of Negative Symptoms (SANS) (Andreasen, 1983), Montgomery Asberg Depression Rating Scale (MADRS) (Montgomery and Asberg, 1979), Young Mania Rating Scale (YMRS) (Young et al., 1978), Social and Occupational Functioning Assessment Scale (SOFAS) (Goldman et al., 1992) and the Global Functioning: Social and Role scales (Cornblatt et al., 2007a). The Brief Assessment of Cognition in Schizophrenia (BACS) was used to measure neurocognition (Keefe et al., 2004). Transition to psychosis was defined using operationalized criteria and assessed with the Comprehensive Assessment of the At-Risk Mental State (CAARMS) (Yung et al., 2005).

### Fatty acid analysis

Fasting erythrocyte fatty acid composition was measured in the phosphatidyl-ethanolamine (PE) fraction through gas chromatography to determine the membrane content of the two major long-chain omega-3 PUFAs (EPA and DHA). The fatty acid biomarker used in the data analysis was the omega-3 index, which is the sum of EPA + DHA. Blood samples were collected at baseline in all participants and stored in EDTA tubes, which were centrifuged at 1500 g for 15 minutes. Samples were subsequently frozen and stored at −80°C.

### Adherence

Adherence to the study medication was assessed monthly for each participant, using the indirect method of a capsule count. The mean adherence rating over the 6-month intervention period was computed, defining non-adherence as <75% capsules taken. In addition, we also assessed the number of returned pills as a continuous measure of non-adherence in a post hoc Cox regression proportional-hazards analysis.

### Statistical analysis

The study sample comprised 285 with fatty acid data at baseline, which was 94% of the total trial sample (*N* = 304). Chi-square tests and independent samples *t*-tests were used to compare adherent and non-adherent participants across both interventions for baseline measures including age, gender, BMI, n-3 fatty acid levels (EPA + DHA, n-3 index), tobacco use, cannabis use, symptoms (BPRS, SANS, MADRS, YMRS), psychosocial functioning (SOFAS, GF-S, GF-R) and neurocognitive functioning (BACS). Kaplan–Meier survival analysis was used to assess differences in time to transition to psychosis between the adherence groups at 12 month follow-up using the log-rank test. An additional post hoc analysis was conducted to examine the 6 months follow-up transition rates. Although the study intervention period ended at 6 months and the follow-up period ended at 12 months (McGorry et al., 2017), approximately 10% of the participants remained in our clinical care after the study follow-up ended. In those individuals, the last known date they have remained non-psychotic was used in the survival analysis. The follow-up duration ranged from 426 to 790 days. In order to determine if non-adherence independently predicted transition to psychosis, we conducted a confirmatory multivariate Cox regression analysis over the entire follow-up period, covarying for factors that were significantly associated with non-adherence. All statistical analyses were two-sided and completed using the IBM SPSS-Statistic software, version 24.0, with alpha = 0.05.

## Results

**Figure 1.**
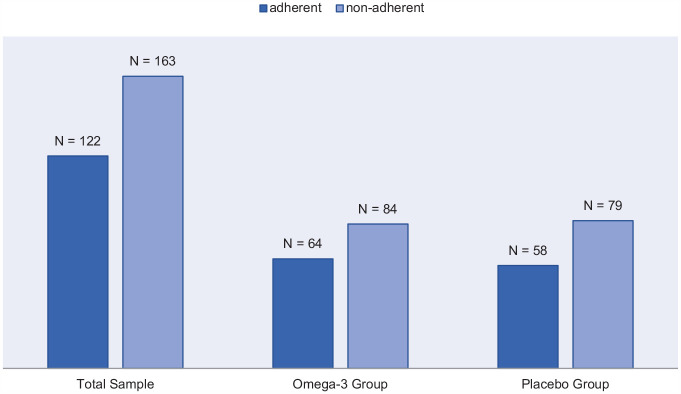
Adherence/non-adherence ratio in NEURAPRO participants.

Of 285 participants with clinical and biomarker data (mean age = 19.1 ± 4.6, male = 45.7%), 163 (57.2%) were non-adherent (omega-3 PUFA: *n* = 84 (56.8%), placebo: *n* = 79 (57.7%) (chi-square = 0.024, *p* = 0.877) (see Figure 1). Biomarker analyses revealed the mean change from baseline to endpoint in the omega-3 index was significantly lower in the non-adherent versus adherent group (−.654, 95% confidence interval [CI]: −1.236, −0.073, *p* = .028) in the omega-3 PUFA sub-group (Amminger et al., 2020), validating results of adherence measure (the pill count).

### Determinants of non-adherence

In univariate analyses comparing adherent and non-adherence groups at baseline, non-adherence was associated with significantly lower baseline omega-3 PUFA levels, longer duration of untreated symptoms (DUS) prior to study entry, a higher proportion of daily tobacco use, a higher proportion of lifetime cannabis use and lower psychosocial functioning (Table 1).

**Table 1.** Baseline characteristics.

	Adherent, *N* = 122	Non-adherent, *N* = 163	*P value
Age	19.18 (4.65)	18.83 (4.38)	0.514
Gender, female	55 (45.1%)	74 (45.4%)	0.958
BMI (SD)	24.01 (5.00)	23.94 (5.82)	0.918
Treatment			0.877
Fish oil	64 (52.5%)	84 (51.5%)	
Placebo	58 (47.5%)	79 (48.5%)	
Omega-3 Index	7.79 (1.94)	7.13 (1.53)	0.003
DUS (days)	1022.4 (1176.93)	724.12 (800.15)	0.012
Smoking (daily)	36/122 (29.5%)	74/163 (45.4%)	0.006
Cannabis (lifetime)	48/122 (39.3%)	88/158 (55.7%)	0.007
BPRS (SD)	40.55 (9.07)	41.82 (10.33)	0.272
SANS (SD)	16.96 (12.40)	19.36 (13.63)	0.121
YMRS (SD)	2.85 (2.62)	3.45 (3.26)	0.089
MADRS (SD)	19.03 (8.62)	19.40 (9.30)	0.723
SOFAS (SD)	56.05 (12.11)	51.29 (11.52)	0.001
GF-*R* (SD)	6.19 (1.56)	5.78 (1.52)	0.027
GF-S (SD)	6.78 (1.20)	6.30 (1.19)	0.001
BACS (SD)	-0.38 (1.43)	-0.53 (1.37)	0.376

Data are mean (SD) or *n* (%).

DUS, duration of untreated symptoms prior to study entry.

* *P* value derived from chi-square or t-test.

### Medication non-adherence and transition to psychosis

**Figure 2.**
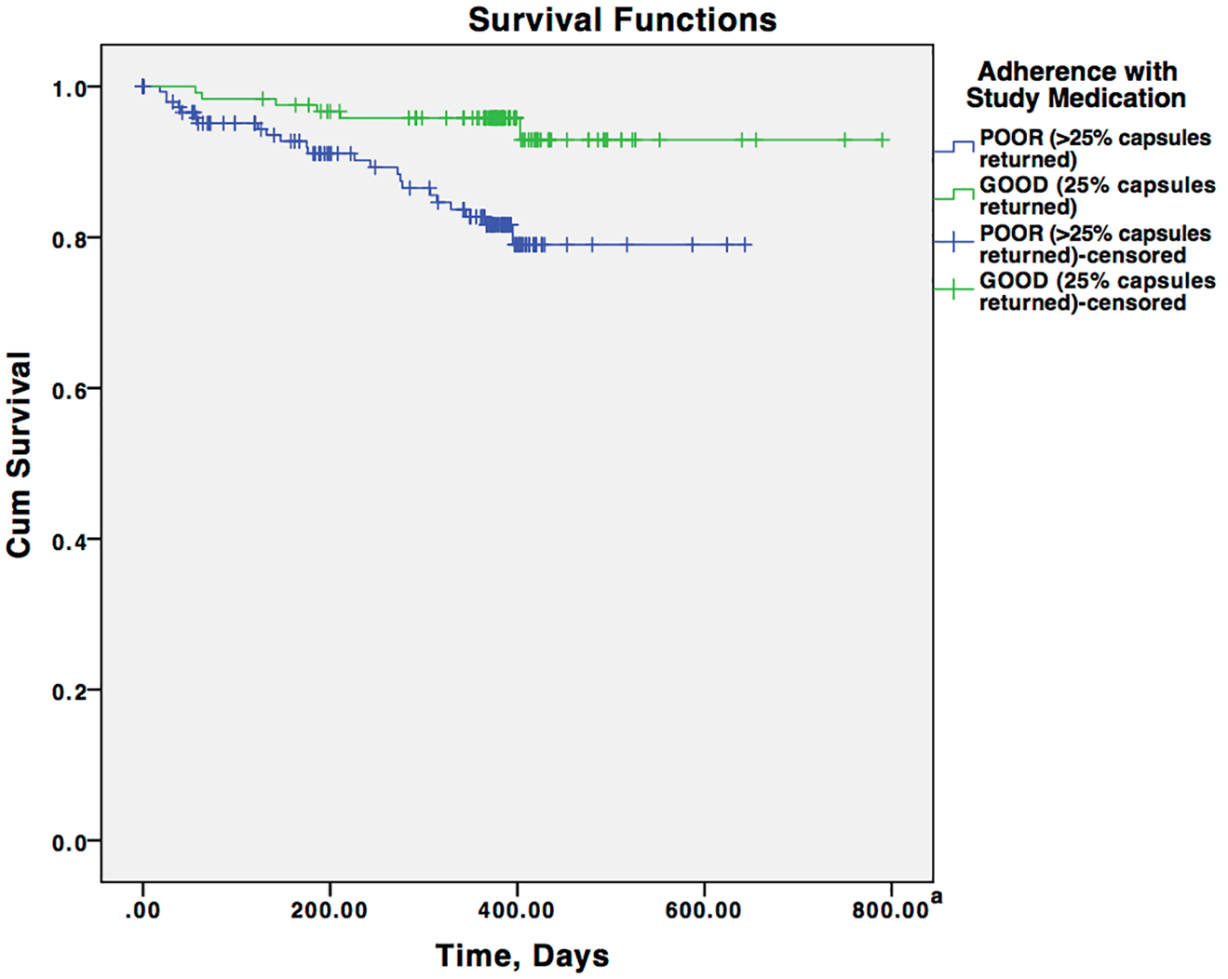
Survival curves for the transition-to-psychosis rate in adherent and non-adherent individual. ^a^Although the study follow-up period ended at 12 months (McGorry et al., 2017), approximately 10% of participants were followed in our clinical care. In those individuals, the last known date of follow-up (range: 426–790 days) was used in the survival analysis.

The Kaplan–Meier cumulative transition rates to psychotic disorder at 12 months were 4.2% (95% CI: 0.7–7.7%) in the adherent group and 17.3% (95% CI: 10.4–24.2%) in the non-adherent group (omega-3 PUFA: 56.8%, placebo: 57.7%, *p* = 0.877). The difference in risk of transition to psychosis between categorically defined adherence groups was 13.1% (95% CI: 5.3–20.8%). The risk of transition to psychosis was significantly lower in the adherent group than in the non-adherent group (log-rank test, chi-square = 10.675, *p* = 0.001) (Figure 2). To further explore the observed group differences, a post hoc analysis was conducted to examine the transition to psychosis rates at 6 months. The results showed transition rates of 2.5% (3 of 122 individuals) in the adherent group and 7.4% (12 of 163 individuals) in the non-adherent group. Consistent with the result of the survival analysis, log-rank test indicated these transition rates were significantly different (chi-square = 5.120, *p* = 0.024).

Furthermore, a Cox regression analysis, covarying for factors significantly associated with categorically defined non-adherence in univariate analyses, corroborated the result of the Kaplan–Meier survival analysis, revealing non-adherence as an independent predictor of transition to psychosis (*B* = 1.452, *p* = 0.005), while among the control variables only longer DUS was significantly related to transition to psychoses, yet with a very small contribution (*B* = 0.000, *p* = 0.044, (Table 2). Finally, a post hoc Cox regression proportional-hazards analysis, in which non-adherence was used as continuous variable also showed that a higher number of returned capsules predicted transition to psychosis (*p* = 0.007).

**Table 2.** Cox regression analysis.

	B	SE	*P* value
Adherence with study medication	1.452	.512	.005
DUS prior to study entry	.000	.000	.044
Smoking (daily or almost daily)	-.312	.471	.508
Cannabis use (Lifetime)	.659	.480	.170
Omega-3 index (T1)	-.046	.120	.701
SOFAS (T1)	.002	.022	.942
Global Functioning—Social (T1)	-.257	.202	.203
Global Functioning—Role (T1)	.275	.182	.132

DUS, Duration of untreated symptoms.

SOFAS, Social and Occupational Functioning Assessment Scale.

T1 = Baseline Assessment.

## Discussion

This study has the following main findings: (1) 57.2% of participants were found to be non-adherent; (2) non-adherence was associated with significantly lower baseline omega-3 index; longer DUS prior to study entry, a higher proportion of daily tobacco use, a higher proportion of life-time cannabis use and lower social functioning at baseline; (3) non-adherence to study medication (omega-3 PUFAs or placebo) was significantly associated with transition to psychosis at 12 months, even after controlling for all other variables that were significantly associated with transition to psychosis, including DUS that had a statistically significant but very small effect in the final model and (4) non-adherence to omega-3 PUFAs or placebo was also significantly associated with risk for transition for psychosis when the number of returned pills was used as a continuous measure of non-adherence.

No previous study has investigated non-adherence in people at UHR for psychosis in a randomized trial. There are various factors that may contribute to the relatively high non-adherence in the NEURAPRO study. The UHR for psychosis group does not meet the diagnostic threshold for psychosis and may not see sufficient reason for taking a medication. This view is supported by findings showing that medication adherence is higher in acute phases of recent onset schizophrenia (Kamali et al., 2006), as opposed to more stable later illness stages (Lincoln et al., 2007). Conversely, the unproven efficacy and benign nature of omega-3 PUFAs may have prompted non-adherence due to doubts about its efficacy. Similarly, given the randomization to either omega-3 PUFAs or placebo, it is possible that some participants assumed they were taking placebo, which might have further lowered their motivation to comply with the treatment regimen. In addition, patients had mostly subthreshold symptomatology, and omega-3 PUFAs were prescribed to prevent worsening, rather than treating suprathreshold and troubling symptoms, or an established disorder. The desire to avoid stigma and not to be viewed as different from their peers could also have contributed to poor adherence (Rüsch et al., 2014, 2015). A lack of parental, family or social support could be a further reason for the observed low adherence rate, since increased medication adherence has previously been observed when family members were trained to be key care supervisors of medication adherence (Farooq et al., 2011). Finally, the non-adherence rate of 57% to an intervention with relatively few side effects may also have been related to the careful assessment of non-adherence and threshold for non-adherence of <75% of prescribed medication.

The finding of a significantly lower baseline omega-3 index in the non-adherent group may provide an initial indication of a possible biological underpinning of non-adherence risk as well as a link between non-adherence and transition to psychosis. While the addition of omega-3 PUFAs to patients independent of their baseline omega-3 PUFA levels and individual biological risk for psychosis did not prove to produce any different effects than placebo, this lack of effect does not invalidate the possibility that inherently lower omega-3 PUFA levels may be associated with a biological vulnerability or environmental effects (e.g. substance use) that may put the patient at higher risk of unhealthy behaviors. For example, lower omega-3 PUFA level have been associated with smoking, alcohol use, higher BMI, lower socioeconomic status and unhealthy lifestyle behaviors (Wagner et al., 2015).

However, more research is needed to determine correlates of non-adherence that adversely affect treatment of psychiatric and other medical conditions.

The significant association between non-adherence and lower social functioning is similar to previous findings of Stentzel and colleagues (Stentzel et al., 2018). They showed that a higher level of global functioning was a significant positive determinant of adherence. Of note, there may be a link between the risk of non-adherence and transition to psychosis with poorer social functioning being related to both as impaired global functioning has been associated with the risk of transitioning to psychosis (Cornblatt et al., 2012; Oliver et al., 2020; Yung et al., 2005). The significant association between cannabis use and non-adherence in this study is in accordance with meta-analytic evidence that cannabis use increased the risk of non-adherence to antipsychotics in patients with psychosis by 150% (Foglia et al., 2017). This speaks to the need to reduce or cease cannabis use to promote better adherence to medications.

The most remarkable result from this study is the significant and independent association between categorically defined non-adherence to treatment (either omega-PUFAs or placebo) and an increased cumulative risk of transition to psychosis at 12 months. The higher rate of transition to psychosis in the group non-adherent to either omega-3 PUFAs or placebo was also evident at 6-month follow-up, as demonstrated in a post hoc analysis. Moreover, the post hoc Cox regression proportional-hazards analysis, using non-adherence as a continuous variable, yielded the same result as the primary analysis that utilized the commonly accepted threshold for non-adherence of <75% of pills taken. This additional analysis corroborated the robustness of the main study result that non-adherence to omega-3 PUFAs or placebo predicted transition to psychosis, independent of whether non-adherence was defined categorically or whether the pill count data were used as a continuous variable.

To our knowledge the association between non-adherence as a medication-independent factor for transition to psychosis has been described in the literature only once before (Cornblatt et al., 2007b). In this naturalistic study of 48 adolescents at clinical high risk (mean age = 15.8 years) who had been followed up for at least 6 months (mean follow-up = 30.5 months), adolescents who became non-adherent to medication were at over four times greater risk for conversion to psychosis then those who are adherent (Cornblatt et al., 2007b). Important to note, this finding is limited by the study design that medication (antipsychotic or antidepressant) was started at clinician’s choice and that non-adherence was highest among teenagers prescribed antipsychotics whether or not they converted.

Notably, the association between non-adherence as a risk factor for poor outcomes independent of the removal of a potentially beneficial medication effect has been reported in the non-psychiatric medical literature. For example, in RCTs, an association between non-adherence to placebo has been associated with a significantly elevated risk for heart failure, coronary heart disease, cardiac arrhythmia, myocardial infarction, hyperlipidemia as well as overall mortality from heart failure or coronary heart disease (Coronary Drug Project Research Group, 1980; Gallagher et al., 1993; Glynn et al., 1994; Granger et al., 2005; Irvine et al., 1999; Obias-Manno et al., 1996; The West of Scotland Coronary Prevention Study Group, 1997). In a meta-analysis of eight studies comparing 16,689 patients with heart failure, coronary heart disease, cardiac arrhythmia, myocardial infarction, hyperlipidemia and good adherence to placebo versus 6371 patient with poor adherence to placebo, poor placebo adherence was associated with a 37% greater risk of poor medical outcome (hazard ratio (HR): 1.47, 95% confidence interval (CI): 1.30–1.67) (Yue et al., 2014). Similar outcomes were reported by two studies not included in this meta-analysis in 1,354 patients with heart failure randomized to placebo, poor placebo adherence was associated with an increased risk of mortality (HR: 1.64, 95% CI: 1.22–2.17) (Pressman et al., 2012). Similarly, in a study of postmenopausal females participating in an estrogen/progestin replacement study to prevent coronary heart disease, total mortality risk was significantly increased among participants randomized to placebo (*n* = 1,375) when adherence was poor (HR: 1.92, 95% CI: 1.08–3.45) (Padula et al., 2012). Taken together, these data suggest that patient factors related to being non-adherent in an RCT predispose to poor outcomes. The exact underlying reasons, however, remain to be elucidated (Enck et al., 2013).

The results of this study need to be interpreted within its limitations. First, the findings from this study are based on post hoc analyses. Since non-adherence and its effect on outcomes in the combined treatment group were not the initial aim and focus of the study, the results are hypothesis-generating in nature. Second, the method of pill counts used to determine non-adherence may not be accurate in all participants (Kane et al., 2013). Although pill count is one of the most commonly used methods to measure adherence in clinical trials, because of the simplicity of this method it may overestimate adherence, as participants can switch medicines between bottles or blister packs and discard pills. Future studies should consider other methods of measuring adherence with increased accuracy in both the active and placebo arms. Although we measured omega-3 PUFA levels in the NEURAPRO study, validating the non-adherence results of the pill count in the active arm, this analysis does not validate assessment of adherence to placebo. Third, since non-adherence was not a primary focus of the study, we did not assess many factors that are potentially relevant for non-adherence behaviors (Enck et al., 2013; Kane et al., 2013). Future studies should include assessments of participant- and illness-related factors, including attitudes and behaviors that have been related to non-adherence risk.

In conclusion, the findings from this study demonstrate the importance of investigating non-adherence in RCTs in the UHR population. Further studies are needed that investigate in greater detail factors associated with non-adherence as well as potential links to the underlying risk for developing psychosis. Knowledge about correlates of non-adherence could also be used to develop strategies to enhance treatment adherence in UHR individuals treated in RCTs or in clinical settings.
